# Integrated network pharmacology and metabolomics to reveal the mechanism of *Pinellia ternata* inhibiting non-small cell lung cancer cells

**DOI:** 10.1186/s12906-024-04574-3

**Published:** 2024-07-11

**Authors:** Fan Feng, Ping Hu, Lei Peng, Lisheng Xu, Jun Chen, Qiong Chen, Xingtao Zhang, Xingkui Tao

**Affiliations:** School of Biological and Food Engineering, Suzhou University, Anhui, 234000 China

**Keywords:** Lung cancer, Metabolomics, Network pharmacology, Metabolites

## Abstract

Lung cancer is a malignant tumor with highly heterogeneous characteristics. A classic Chinese medicine, *Pinellia ternata* (PT), was shown to exert therapeutic effects on lung cancer cells. However, its chemical and pharmacological profiles are not yet understood. In the present study, we aimed to reveal the mechanism of PT in treating lung cancer cells through metabolomics and network pharmacology. Metabolomic analysis of two strains of lung cancer cells treated with *Pinellia ternata* extracts (PTE) was used to identify differentially abundant metabolites, and the metabolic pathways associated with the DEGs were identified by MetaboAnalyst. Then, network pharmacology was applied to identify potential targets against PTE-induced lung cancer cells. The integrated network of metabolomics and network pharmacology was constructed based on Cytoscape. PTE obviously inhibited the proliferation, migration and invasion of A549 and NCI-H460 cells. The results of the cellular metabolomics analysis showed that 30 metabolites were differentially expressed in the lung cancer cells of the experimental and control groups. Through pathway enrichment analysis, 5 metabolites were found to be involved in purine metabolism, riboflavin metabolism and the pentose phosphate pathway, including D-ribose 5-phosphate, xanthosine, 5-amino-4-imidazolecarboxyamide, flavin mononucleotide (FMN) and flavin adenine dinucleotide (FAD). Combined with network pharmacology, 11 bioactive compounds were found in PT, and networks of bioactive compound–target gene–metabolic enzyme–metabolite interactions were constructed. In conclusion, this study revealed the complicated mechanisms of PT against lung cancer. Our work provides a novel paradigm for identifying the potential mechanisms underlying the pharmacological effects of natural compounds.

**Supplementary Information:**

The online version contains supplementary material available at 10.1186/s12906-024-04574-3.

## Introduction

Lung cancer is a malignant tumor with highly heterogeneous characteristics. Lung cancer has the highest prevalence and mortality rate of all malignant cancers both in China and worldwide [1]. According to the latest data from GLOBOCAN2020, the incidence and mortality of lung cancer in China account for 37.0% and 39.8% of the world’s total, respectively, showing an increasing trend in recent years [2]. Therefore, preventing and treating lung cancer is a major challenge for preventing and controlling malignant tumors. At present, effective treatments for lung cancer mainly rely on the development and use of targeted drugs, but their clinical benefits are still limited. Therefore, the clinical prevention and treatment of lung cancer by combining multicomponent traditional Chinese medicine is highly valuable [3–5]. Many studies have shown that traditional Chinese medicine induces cell apoptosis and inhibits cell proliferation, which can alleviate symptoms, inhibit tumor development, prolong survival and improve the quality of life of patients [6–8]. Due to the lack of support for large-scale clinical trials based on evidence-based medicine and the limitations of statistics, traditional Chinese medicine is often used as an adjuvant treatment for lung cancer patients. Many published reports have shown the results of combining TCM with chemotherapy in lung cancer patients [9–11], and several natural compounds from traditional Chinese medicine formulas, such as resveratrol, curcumin, and berberine, have been shown to exhibit anticancer effects that inhibit the development, proliferation, angiogenesis, and metastasis of lung cancer [12]. Unlike Western medicine of “one target, one drug”, TCM theory emphasizes the concept of the integrity of the whole human body. Due to the complexity of its components, conventional pharmacological approaches for experimentally identifying the unique mechanism of action may not be suitable for TCM research [13]. With the rapid development of bioinformatics, newly emerging network pharmacology based on large databases has become a useful tool for the detailed characterization of complex drug system mechanisms from the molecular level to the pathway level [14]. Network pharmacology conforms to the key ideas of the holistic philosophy of TCM. As a state-of-the-art technology, this method updates the research paradigm from the current “one target, one drug” mode to a new “network target, multicomponent” mode [15–17].

Most lung cancer originates from malignant bronchial mucosal epithelium, with a small portion caused by bronchial alveolar epithelium or adenoid lesions. Some of lung cancer’s typical symptoms are difficulty breathing, coughing and the expectoration of sputum [18]. Therefore, traditional Chinese medicine experts in the past have paid attention to the use of expectorant products in treatment. *Pinellia ternata*, recorded in “Shen Nong’s Materia Medica”, has a long history in treating cough. However, the chemical and pharmacological foundations of *Pinellia ternata* in inhibiting human cancers, especially lung cancer, have not been globally evaluated with appropriate approaches. Nonetheless, network pharmacology is limited by the use of a single computational method that relies on public databases. Network pharmacology alone could only predict the possibility of compound-target combinations and pathway analysis [16]. Metabolomics, the simultaneous analysis of a large pool of endogenous metabolites, has been applied in many fields, including the diagnosis and treatment of diseases, biomarker discovery, and the exploration of disease pathogenesis [19, 20]. Therefore, we integrated metabolomics with network pharmacology to analyze the mechanism of action of *Pinellia ternata*. This strategy is expected to help researchers better understand the therapeutic principles of natural *Pinellia ternata* compounds in the treatment of lung cancer.

**Fig. 1:**
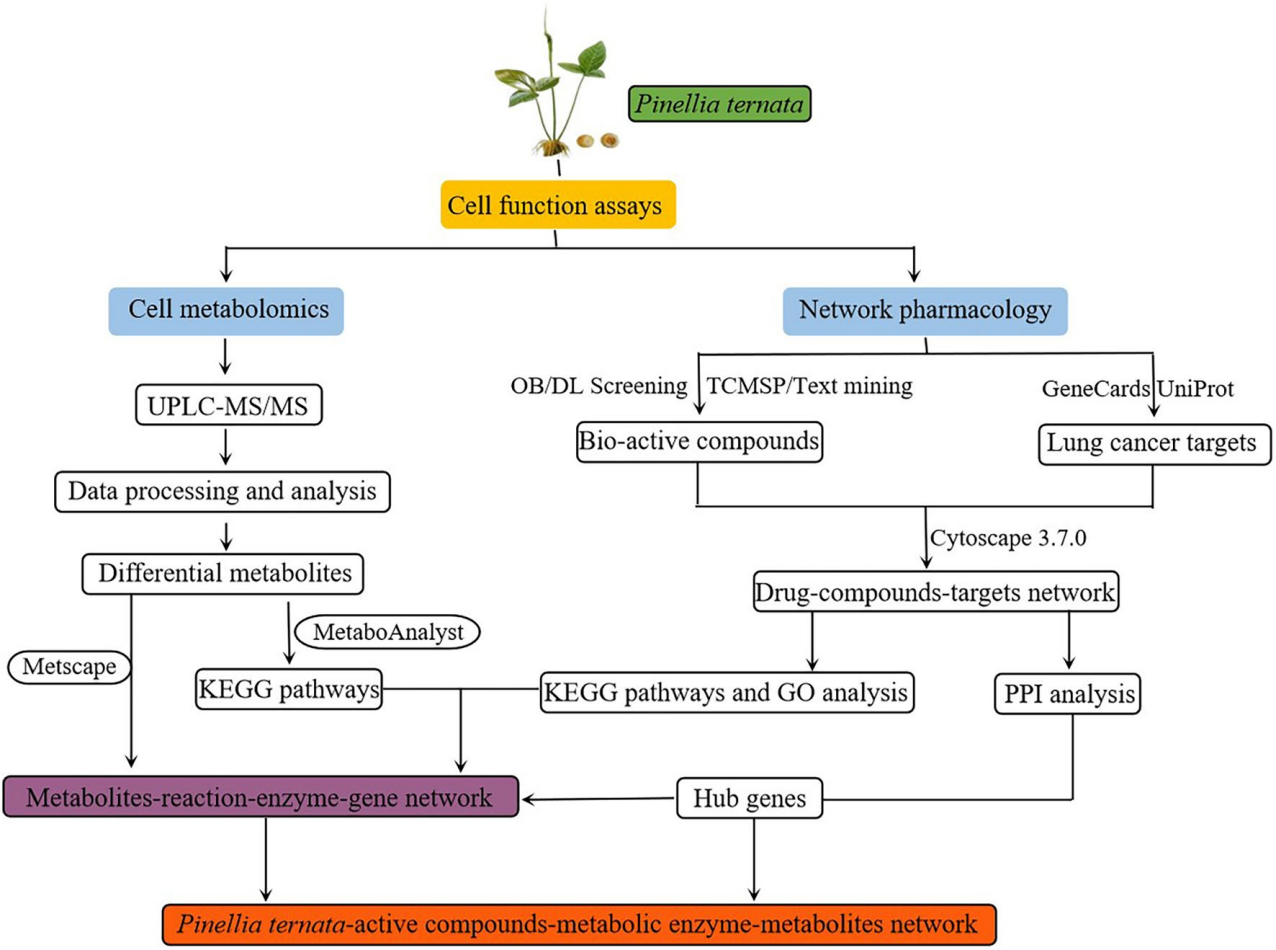
The technical strategy of the current study

In the present study, we used computational tools and resources to investigate the effects of the pharmacological network of *Pinellia ternata* on lung cancer to predict its active compounds and potential protein targets and pathways. In addition, in vitro experiments were also conducted to validate the potential underlying mechanism of *Pinellia ternata* in lung cancer, as predicted by a network pharmacology approach. Moreover, metabolomic analysis of lung cancer cells revealed synergistic metabolic mechanisms in terms of metabolites and metabolic pathways. Subsequently, the targets from network pharmacology and the metabolites from cell metabolomics were jointly analyzed to filter crucial metabolic pathways via MetaScape. The detailed technical strategy of the current study is shown in Fig. 1.

## Materials and methods

### Cell experiments

#### Reagents

The tuber of *Pinellia ternata* was collected from our experimental field at Suzhou University and was authenticated by Professor Jianping Xue at the College of Life Sciences, Huaibei Normal University. The geographical location of the experimental field of Suzhou University was 117° E and 34° N. The collection of tubers of *Pinellia ternata* was approved by Zhang Xingtao, the dean of Suzhou University, and our coauthor. Chemical reagents such as ethanol and methanol (analytical grade) were purchased from China National Pharmaceutical Group Co., Ltd.

#### Preparation of herb extracts

The extracts of *Pinellia ternata* were prepared as follows. Two kilograms of *Pinellia ternata* tubers were soaked in 70% ethanol (1:8, w/v) for 2 h and extracted twice with 70% ethanol for 2 h. Later, the extracts were concentrated in vacuo, lyophilized into powder and stored at -80 °C (the drug extraction ratio was 13.8%).

#### Cell culture and cell viability assay

The human normal cell Line BEAS-2B, and non-small cell lung cancer cell Lines A549 and NCI-H460 were purchased from Wuhan Pricella Biotechnology Co., Ltd. (item code: CL-0016, CL-0299) and used for subsequent experiments. The cells were cultured in DMEM supplemented with 10% FBS, 100 U/mL penicillin, and 100 mg/mL streptomycin and maintained at 37 °C in a humidified chamber with 5% CO_2_.

The human normal lung cell and lung cancer cells (5,000 cells/well) were seeded in 96-well plates and incubated for 24 h. After pretreatment with different concentrations of PTE (0, 0.0125, 0.025, 0.05, 0.10, 0.20, 0.40, and 0.80 µg/µL) for 48 h, 10 µL of 3-(4,5-dimethylthiazol-2-yl)-2,5-diphenyltetrazolium bromide solution (MTT, 5 mg/ml; Sigma, USA) was added to each well, and then the cells were cultured at 37 °C for another 4 h. Then, the supernatants were discarded, and 100 µL of DMSO was added to each well. The absorbance was measured at 490 nm using a Multiskan MS microplate reader (Labsystems, Finland). The IC_50_ of PTE in lung cancer cells was calculated by using GraphPad Prism 9 software.

#### Cell wound-healing and transwell invasion assays

For the transwell invasion assay, millicell cell cultures inserted in 24-well plates were pretreated with 100 µL of cold Matrigel (BD Biosciences, USA, diluted 1:4 with cold PBS) for 2 h at 37 °C. Lung cancer cells (1 × 10^5^ cells/well) were seeded in a chamber with 200 µL of serum-free DMEM at 37 °C and then incubated with or without PTE at the IC_50_ for 24 h. The invaded cells were fixed with 4% paraformaldehyde for 30 min, stained with crystal violet solution for 2 h and then counted with a light microscope.

For the wound-healing assay, lung cancer cells were incubated in 6-well plates at 100% confluence. A denuded area was scraped from the cell monolayer using a plastic pipette tip. The medium was removed, and the monolayer was washed 3 times with PBS. Then, medium with or without PTE at the IC_50_ was added to each well, and cell movement into the wound area was assessed after 24 h of incubation under a microscope.

### UPLC‒MS metabolomic analysis

#### Experimental grouping and sample preparation

A549 and NCI-H460 cells were removed, cultured in 100 mm Petri dishes and cultured overnight to allow the cells to adhere to the wall. The cells were divided into a blank control group and an intervention group and treated with PTE at a concentration of 0 or the IC50, with 6 parallel samples in each group. Therefore, the treated A549 and NCI-H460 cells were divided into four main groups: Control-A, PTE-A, Control-N, and PTE-N. After 48 h, the Petri dish was washed three times with precooled PBS and then digested with trypsin for 1–2 min. The suspension was centrifuged at 1000 r/min for 5 min, the supernatant was discarded, and the cells were collected as samples.

The samples stored at -80 °C were thawed on ice. A 500 µL solution (methanol: water = 4:1, V/V) containing an internal standard was added to the cell sample and vortexed for 3 min. The sample was placed in liquid nitrogen for 5 min and on dry ice for 5 min and then thawed on ice and vortexed for 2 min. This freeze‒thaw cycle was repeated three times in total. The sample was centrifuged at 12,000 rpm for 10 min (4 °C). Then, 300 µL of the supernatant was collected and stored at -20 °C for 30 min. The sample was then centrifuged at 12,000 rpm for 3 min (4 °C). A 200 µL aliquot of the supernatant was transferred for LC‒MS analysis. The pooled quality control (QC) samples were made by mixing 10 µL aliquots from each sample (one per six samples).

#### UPLC-QTOF/MS analysis

All samples were acquired by the LC‒MS system following the manufacturer’s instructions. The analytical conditions were as follows: UPLC: column, Waters ACQUITY UPLC BEH C18 1.8 μm, 2.1 mm * 100 mm; column temperature, 40 °C; flow rate, 0.4 mL/min; injection volume, 2 µL; and solvent system, water (0.1% formic acid): acetonitrile (0.1% formic acid). The column was eluted with 5% mobile phase B (0.1% formic acid in acetonitrile) at 0 min, followed by a linear gradient to 90% mobile phase B (0.1% formic acid in acetonitrile) over 11 min, held for 1 min, and then returned to 5% mobile phase B within 0.1 min, held for 1.9 min, and then rapidly returned to the starting conditions.

The data were acquired in information-dependent acquisition (IDA) mode using Analyst TF 1.7.1 Software (Sciex, Concord, ON, Canada). The source parameters were set as follows: ion source gas 1 (GAS1), 50 psi; ion source gas 2 (GAS2), 50 psi; curtain gas (CUR), 35 psi; temperature (TEM), 550 °C, or 450 °C; declustering potential (DP), 60 V, or − 60 V in positive or negative mode, respectively; and ion spray voltage floating (ISVF), 5000 V–− 4000 V in positive or negative mode, respectively.

#### Data analysis

The original data file acquired by LC‒MS was converted into mzML format by ProteoWizard software. Peak extraction, peak alignment and retention time correction were performed by the XCMS program. The “SVR” method was used to correct the peak area. The peaks with detection rates lower than 50% in each group of samples were discarded. After that, metabolic identification information was obtained by searching the laboratory’s self-built database, integrated public database, AI database and metDNA. SIMCA-P 14.1 software (Umetrics, Sweden) was used to conduct principal component analysis (PCA), partial least-squares discriminant analysis (PLS-DA), and orthogonal partial least-squares (OPLS) analysis of the normalized data. Based on the VIP values (VIP > 1) and t tests (*P* < 0.05), the differentially abundant metabolites were selected between the control group and the model group and identified according to the following online databases: mzCloud (https://www.mzcloud.org/), HMDB (http://www.hmdb.ca), ChemSpider (http://www.chemspider.com), and KEGG (http://www.kegg.jp) [21]. The Venn diagram was drawn according to the guidance method of online software (https://cloud.metware.cn/#/user/login). Pathway analysis was conducted with MetaboAnalyst [22]. Parameters (*p* value < 0.05) were used as indices to determine the most relevant pathways.

### Network pharmacology analysis

#### Bioactive component screening

Information on the *Pinellia ternata* compounds was obtained from databases such as TCMSP (http://tcmspw.com/) [23]. The active compounds were filtered by integrating oral bioavailability (OB) (≥ 30%) and drug similarity (DL) (≥ 0.18) [24]. In addition, compounds with definite pharmacological effects, even those with low OB or DL values, were selected for further research.

#### Target protein prediction of drug components in Pinellia ternata

The protein targets of the active substances in *Pinellia ternata* were retrieved from the TCMSP database by using the filter search bar of the related targets of the compound component. Moreover, the annotated genome database platform Gene Cards, the protein database UniProt, and the online database KOBAS were used to query the human gene names corresponding to the target proteins.

#### Construction of the protein–protein interaction (PPI) network and screening of its core targets

The String database (https://string-db.org/Version 10.5) was logged online, and the search mode “multiple protein” box was used. Then, common target proteins of *Pinellia ternata* and lung cancer were entered into the String database. The search criteria for the species condition were human (*Homo sapiens*); the common target name was converted; the PPI score was set to > 0.7, and a visual interaction map of the PPI relationship network was obtained. The free proteins that appeared outside the network were manually hidden, and the PPI interaction protein relationship map was exported. According to the node degree values, the key core genes of the protein interaction network were screened out.

#### Pathway enrichment analysis

To explore the combination mechanisms of *Pinellia ternata* against lung cancer, pathway enrichment was performed using the DAVID Bioinformatics Resources 6.8 server [25], and GO and KEGG pathway enrichment analyses of drugs, key chemical components, and disease targets were carried out. Pathways with p values less than or equal to 0.05 were selected.

#### Network construction

Combined with the identification and screening of drug target proteins in step 2.3.3, the lung cancer target proteins were mapped to each other to obtain common target proteins, and then, the related information of the drug active ingredient and the common target proteins were imported into Cytoscape 3.7.1 software for data processing. The visual network of drug-bioactive component-disease targets was constructed and obtained. Among them, nodes were used to represent key chemical components and disease targets, and solid lines with arrows were used to represent the interactions between nodes.

#### Joint pathway analysis

The targets from network pharmacology and the metabolites from cell metabolomics were jointly analyzed to select crucial metabolic pathways by MetaboAnalyst [26].

### Western blotting

Lung cancer cells were inoculated in 6-well plates (5 × 10^5^ cells/well). After incubation overnight, cells were treated with or without PTE for 48 h. The cells were harvested using a micro scraper (Corning). The expression levels of GAPDH, p-PI3K p-AKT, MMP9, HIF-1α, TGF-β, BCL-2, and AKT were examined by immunoblotting. In short, the whole cell extracts were lysed on ice with RIPA buffer supplemented with phosphatase inhibitor (1 mM NaF and 1 mM Na_3_VO_4_) and proteinase inhibitor (0.5% aprotinin, 0.5% leupeptin, and 1% PMSF) for 30 min. Then the lysates were centrifuged at 14,000 rpm at 4℃ for 10 min. The protein concentration was measured using bovine serum albumin (BSA; Sigma, MO, USA) as detected using bovine serum albumin as the standard. The same amount of protein in each sample was resolved by sodium dodecyl sulfate-polyacrylamide gel electrophoresis (SDS-PAGE), and transferred onto a polyvinylidene fluoride membrane (PVDF, Biorad, USA). Subsequently, the membrane was blocked with 5% BSA at room temperature in Tris buffered saline-Tween 20 buffer (TBST: 1% Tween 20, 10 mmol/L Tris, 150 mmol/L NaCl, pH 7.4) for 2 h. Then the blots were incubated with primary antibodies (Abcam, UK) overnight at 4ºC. After washing three times with TBST buffer, the blots were incubated with the secondary antibody (Abcam, UK) at room temperature for 2 h. Immunoreactivity was measured using advanced ECL assay kit (GE Healthcare, UK) and visualized using a chemiluminescence imaging system.

### Statistical analysis

Each independent experiment was repeated at least three times. Statistical analysis between two groups was performed by using Student’s t test with SPSS software (SPSS Inc., USA). Variance analysis between multiple groups followed by Tukey’s test was used to calculate the statistical significance of the differences. Multiple groups of normalized data were analyzed using one-way ANOVA. The data are shown as the mean ± standard deviation. Unless otherwise specified, a p value less than 0.05 was considered to indicate a statistically significant difference.

## Results

### *Pinellia ternata* inhibited the proliferation of lung cancer cells

**Fig. 2:**
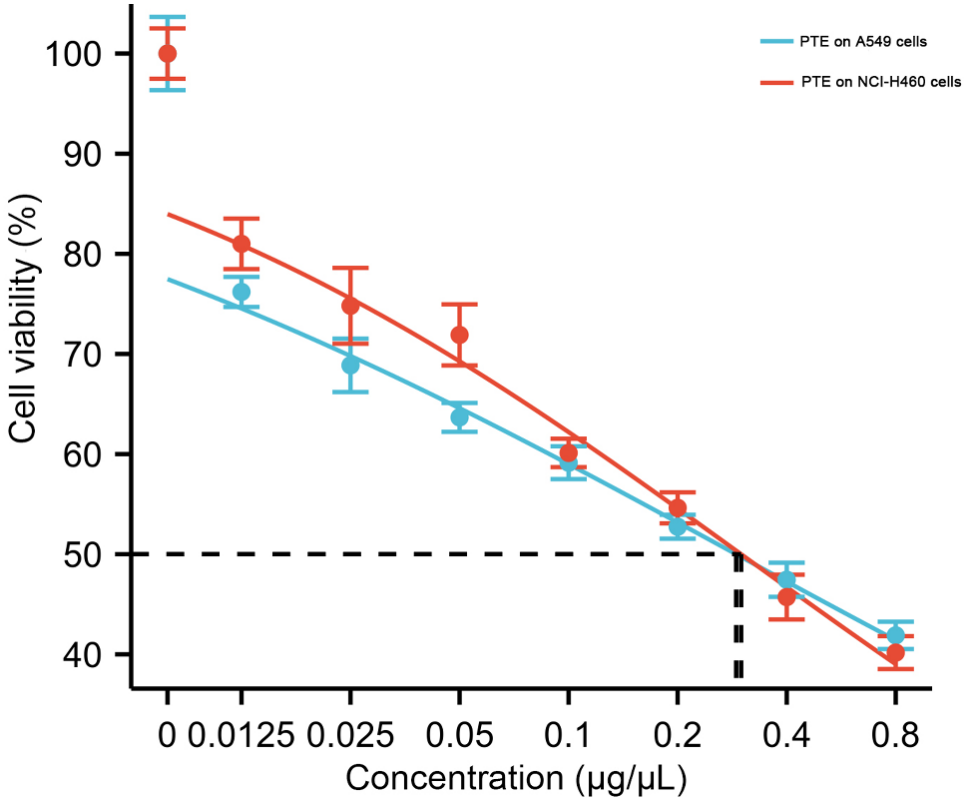
Effects of PTE at different concentrations on the viability of lung cancer A549 and NCI-H460 cells

To validate the anti-proliferative effect of PTE on lung cancer cells, BEAS-2B, A549 and NCI-H460 cells were treated with *Pinellia ternata* extracts at different concentrations for 48 h (Supplementary File S1). The results showed that PTE has little effect on the proliferation of normal lung cells BEAS-2B (Supplementary File S2), but the percentage of inhibited A549 and NCI-H460 cells increased significantly with increasing PTE concentration, indicating that PTE had a significant inhibitory effect on the growth of lung cancer cells (Fig. 2). The IC50 values measured after PTE treatment of A549 and NCI-H460 cells were 0.29 and 0.30 µg/µL, respectively. For the convenience of subsequent experiments, 0.30 µg/µL PTE was selected for treatment of both cell lines.

### *Pinellia ternata* inhibited the migration and invasion of lung cancer cells

**Fig. 3:**
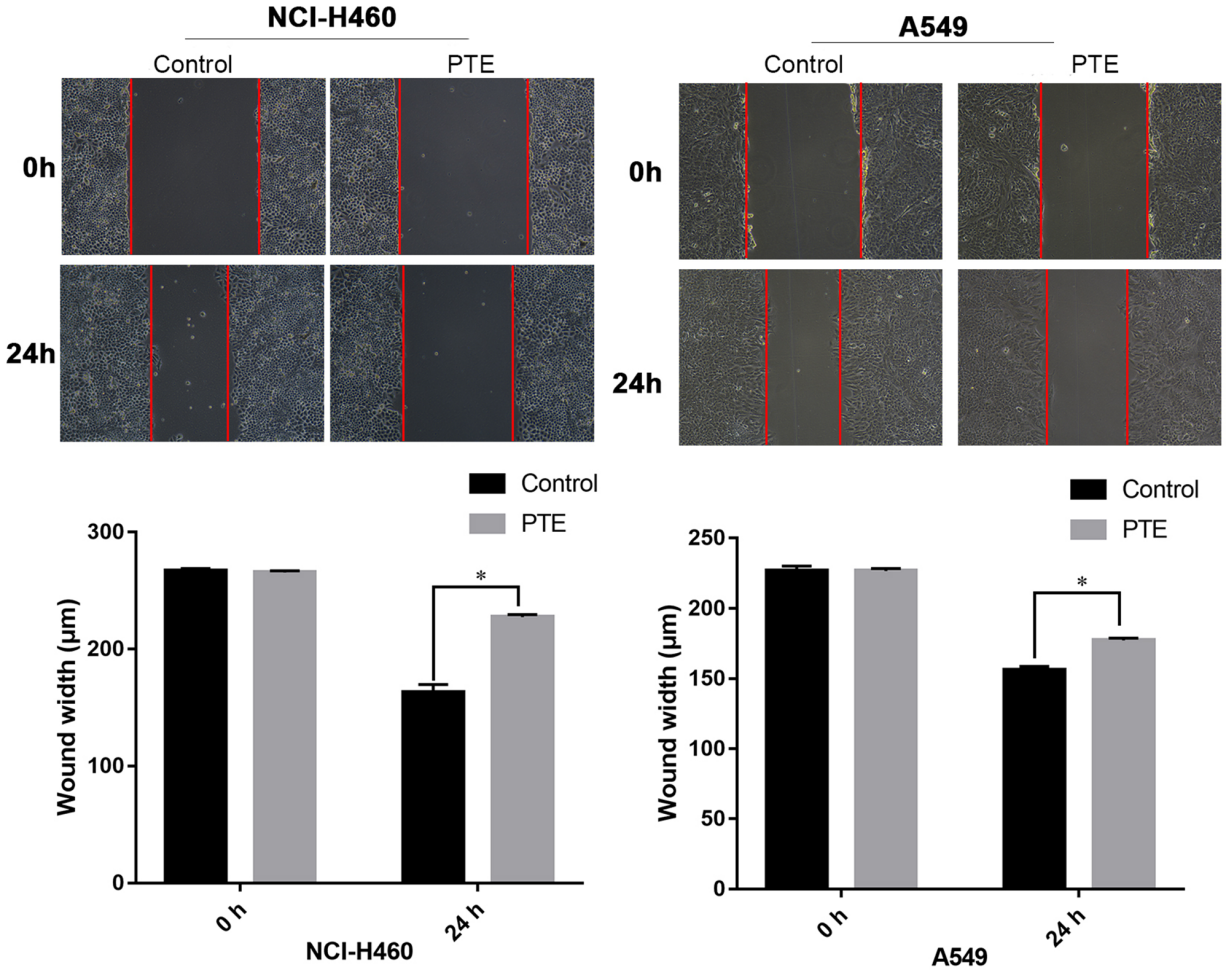
Effects of PTE on the migration of A549 and NCI-H460 cells. * represents *p* < 0.05 compared with the control group

**Fig. 4:**
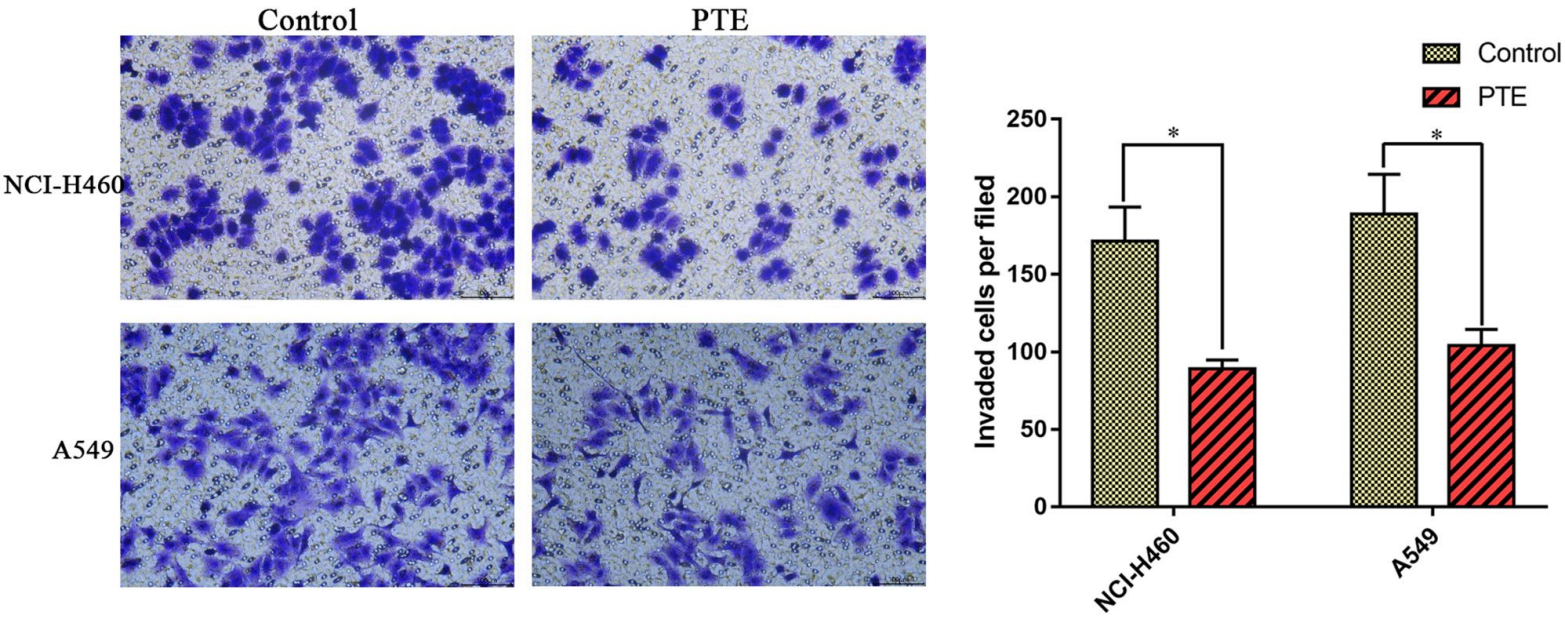
Effects of PTE on the invasion of A549 and NCI-H460 cells. * represents *p* < 0.05 compared with the control group

To investigate the effects of PTE on lung cancer cells in vitro, cell migration and invasion experiments were conducted. As shown in Fig. 3, the wound-healing assay indicated that migration was slower in A549 and NCI-H460 cells treated with PTE, which showed that PTE at a concentration of 0.30 µg/µL obviously inhibited lung cancer cell migration. Moreover, compared with that in the control group, the invasion rate was lower in the PTE-treated A549 and NCI-H460 cell groups (Fig. 4). These experiments indicated that PTE could significantly reduce cell migration and invasion in vitro.

### Results of the metabolomics analysis of lung cancer cells treated with *Pinellia ternata*

#### Multivariate data analysis

**Fig. 5:**
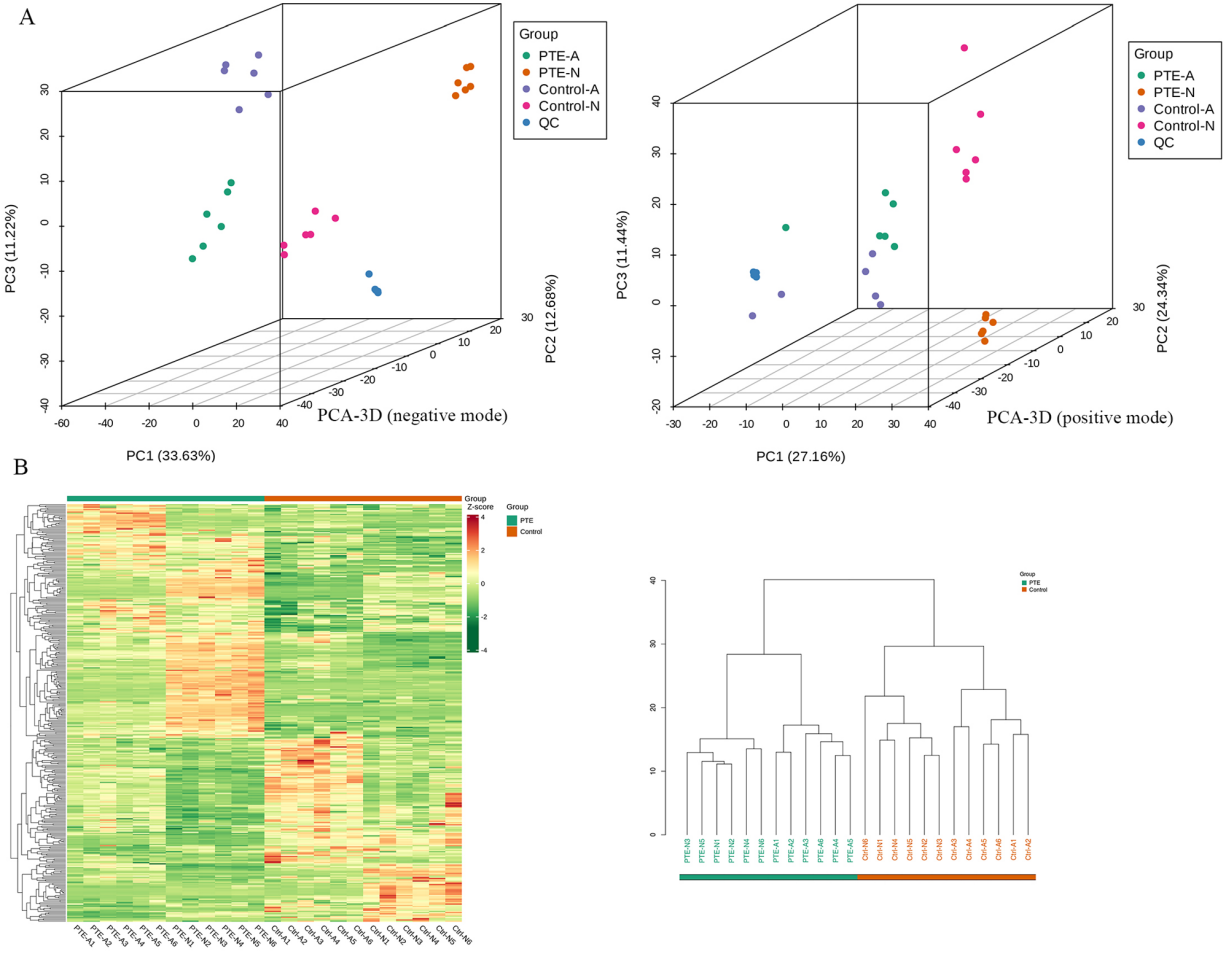
Principal component analysis and cluster analysis of overall metabolites. (**A**) PCA-3D score chart of overall metabolites from LC–MS data in positive and negative mode. (**B**) Overall cluster diagram of the metabolites. The data were analyzed via the Pearson correlation method after mean centering and unit variance scaling

The typical peak intensity chromatograms of the A549 and NCI-H460 lung cancer cell samples were analyzed in both positive and negative modes (Fig. S1). The PCA score plots indicated that the model group was separated from the control group (Fig. 5A). Moreover, a quality control (QC) group was generated, which indicated that the instrument was stable (Fig. 5A). Pearson correlation analysis was conducted on the QC samples, and the correlation between the QC samples was greater than 0.99 (Fig. S2), indicating good stability and high data quality throughout the testing process. As shown in Fig. 5A, significant separations between the PTE group and the control group were observed in the PCA score 3D plots, suggesting that metabolic disturbances could be obviously induced by PTE treatment. Compared with those in the control group, the metabolic changes in the NCI-H460 group were more obvious than those in the A549 group. Further cluster analysis revealed that, after PTE treatment, there were significant differences in metabolites among different lung cancer cell lines, with smaller individual differences within the same group (Fig. 5B).

#### Identification of differential endogenous metabolites

**Fig. 6:**
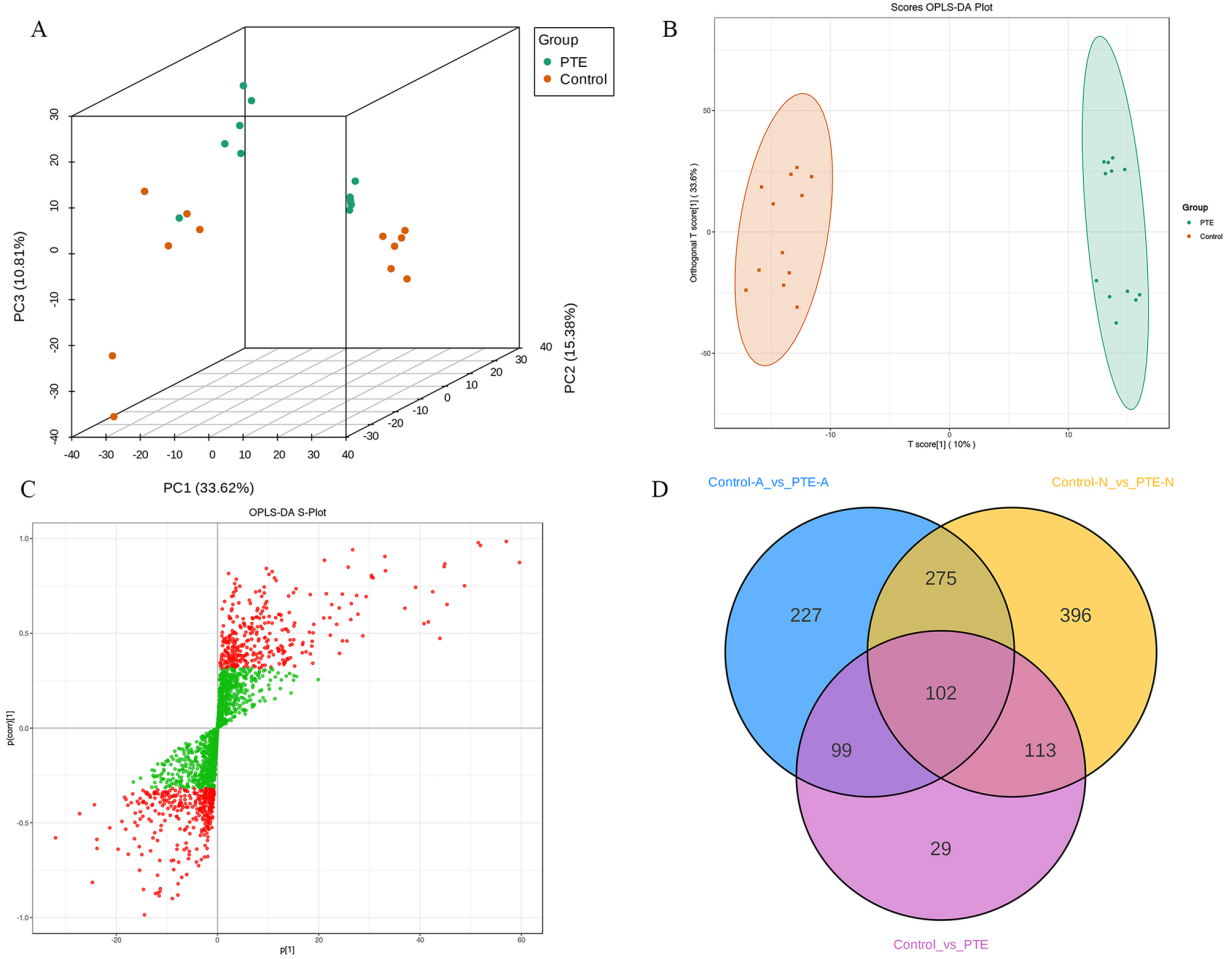
Identification of differential endogenous metabolites. (**A**) PCA score plots, (**B**) OPLS-DA score plot, (**C**) S-plot of OPLS-DA, (**D**) Venn diagrams of the potential metabolites associated with PTE treatment of lung cancer cells

The levels of 2068 metabolites in the lung cancer cells of the 4 groups were determined after data preprocessing (Supplementary File S3). To identify the potential metabolites that contributed to the metabolic differences, we performed PCA (Fig. 6A), OPLS-DA (Fig. 6B) and ANOVA followed by FDR. The OPLS-DA model showed good separation, with high R^2^Y (R^2^Y = 0.991, *p* < 0.005) and Q^2^ (Q^2^ = 0.893, *p* < 0.005) (Fig. S3), indicating good explanatory ability of the sample classification information and cross-validated predictive capability. The S plots of OPLS-DA were constructed based on the VIP values (Fig. 6C), which revealed the variety of metabolites.

Based on VIP > 1 and *p* < 0.05, 703 metabolites in A549 lung cancer cells were differentially expressed between the PTE-A and Control-A groups, 886 metabolites in NCI-H460 lung cancer cells were differentially expressed between the PTE-N and Control-N groups, and 343 metabolites in lung cancer cells were differentially expressed between the PTE group (PTE-A and PTE-N) and Control group (Control-A and Control-N) (Supplementary File S4–6). Venn diagram analysis revealed 102 common metabolic differences between the PTE-treated lung cancer cell group and the control group (Fig. 6D).

#### Metabolic pathway analysis

**Fig. 7:**
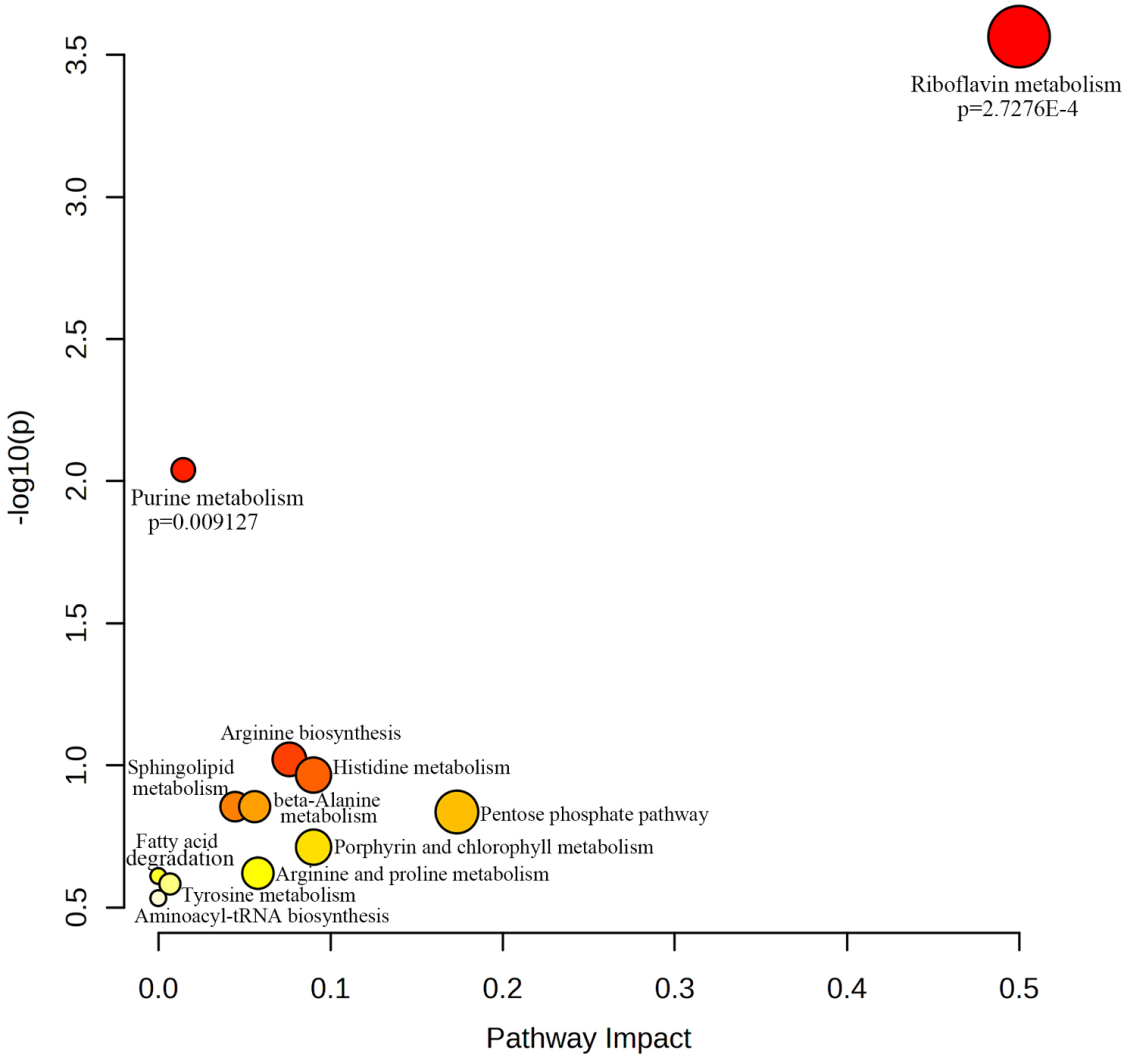
Metabolic pathway analysis. The horizontal axis represents the influence value of the pathway, and the vertical axis represents the significance impact value of the signaling pathway

The 102 differentially abundant metabolites were imported into the KEGG compound database to search for their corresponding KEGG ID, and only 30 metabolites could be identified with the KEGG ID (Supplementary File S7). These metabolites were subsequently imported into MetaboAnalyst to explore the potential anti-lung cancer mechanisms of *Pinellia ternata*, and 12 metabolites were matched for further pathway analysis. As shown in Fig. 7, when the p value was less than 0.05, 2 pathways, namely, purine metabolism and riboflavin metabolism, were significantly affected in the lung cancer cells (Fig. S4). The metabolites related to these pathways were D-ribose 5-phosphate, xanthosine, 5-amino-4-imidazolecarboxyamide, FMN and FAD. Interestingly, D-ribose 5-phosphate was also involved in the pentose phosphate pathway (Table 1).

**Table 1:** Differential metabolites related to the inhibitory effect of *Pinellia ternata* on lung cancer cells detected by UPLC-MS

NO	Metabolites	TR (min)	m/z	Formula	VIP	*P*	Fold change	Trend	KEGG ID	Scan mode
1	Flavin adenine dinucleotide (FAD)	3.00	785.15	C_27_H_33_N_9_O_15_P_2_	1.58	1.51E-02	1.38	↑	C00016	+
2	Flavin mononucleotide (FMN)	3.01	456.10	C_17_H_21_N_4_O_9_P	2.29	2.28E-05	1.39	↑	C00061	+
3	5-Aminoimidazole-4-carboxamide	2.09	126.05	C_4_H_6_N_4_O	1.56	1.15E-02	1.17	↑	C04051	–
4	Xanthosine	1.64	284.07	C_10_H_12_N_4_O_6_	1.23	4.61E-02	2.03	↑	C01762	–
5	D-ribofuranose 5-phosphate	0.86	230.02	C_5_H_11_O_8_P	1.68	1.51E-02	0.53	↓	C00117	–

TR: Retention Time; UPLC-MS: Ultra-Performance Liquid-Chromatography - Mass Spectrometry; VIP: Variable Important in Projection

### Results of the network pharmacological analysis of *Pinellia ternata*

A total of 116 chemical components of *Pinellia ternata* were retrieved from the TCMSP database via keyword screening. By setting the inclusion criteria at DL ≥ 0.18 and OB ≥ 30%, a total of 13 candidate compounds were retrieved (Table S1). Among them, 24-ethylcholest-4-en-3-one, β-sitosterol, poriferast-5-en-3beta-ol, cavidine, baicalin, stigmasterol and other components have drug-like properties of more than 75%, suggesting that these chemical components may play a key regulatory role in the function of this medicine in the human body. A total of 175 human target genes were matched from the 13 compound components identified through searches of the TCMSP database. After deduplication, 99 target genes were ultimately obtained.

**Fig. 8:**
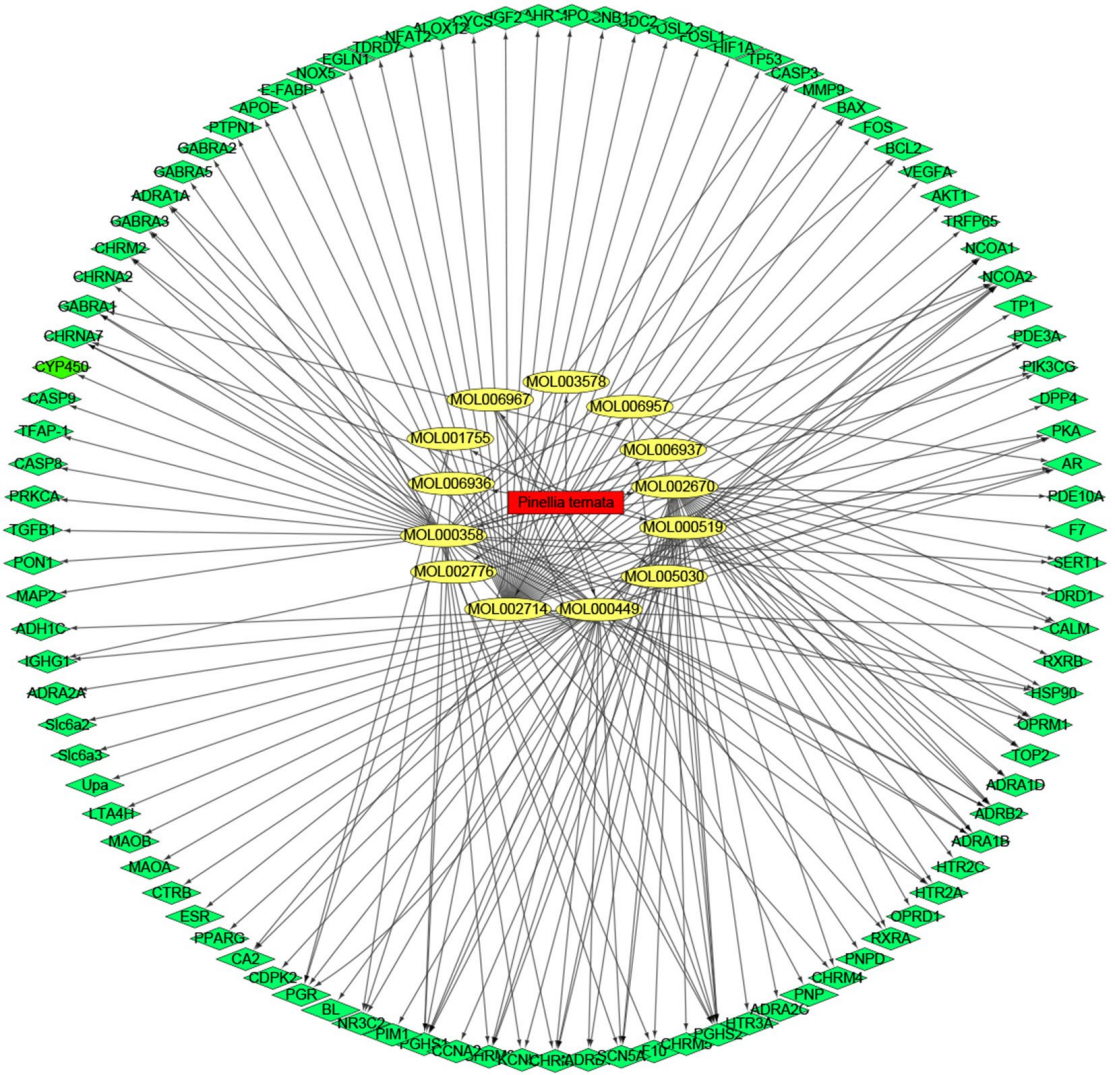
The drug-bioactive component-disease target network for *Pinellia ternata* in lung cancer. The red nodes represent *Pinellia ternata* drugs; the yellow nodes represent candidate active compounds, and the green nodes represent potential protein targets. The edges represent the interactions between these nodes

Then, 22,400 target genes of lung cancer were obtained from the Gene Cards database by using “lung cancer” as the screening keyword, and a total of 4559 genes were obtained by setting the correlation score greater than or equal to 6. By combining the 99 screened drug targets for mutual mapping, 99 common target genes were obtained. The relationships between these 13 compounds and 99 target proteins were analyzed by Cytoscape software to construct a network visualization map of drugs, key chemical components, and disease targets, as shown in Fig. 8.

### Integrated analysis of metabolomics and network pharmacology

**Fig. 9:**
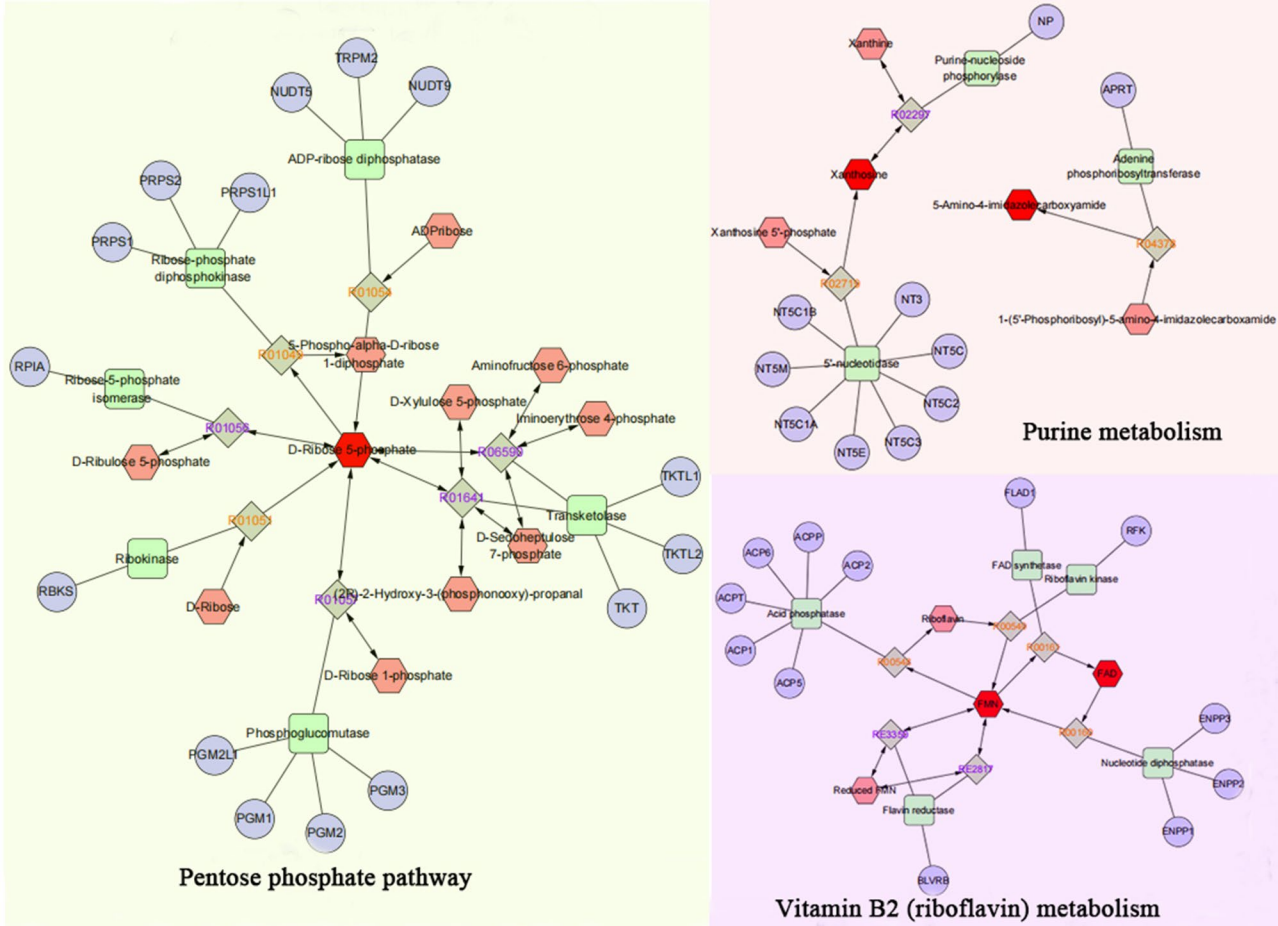
The compound–reaction–enzyme–gene networks of the key metabolites. The red hexagons, gray diamonds, green rectangles and purple circles represent the active compounds, reactions, proteins and genes, respectively. The key metabolites, proteins and genes are magnified

To obtain a comprehensive view of the mechanisms of *Pinellia ternata* against lung cancer cells, we constructed an interaction network based on metabolomics and network pharmacology. Differentially abundant metabolites were imported into the MetScape plugin in Cytoscape to construct metabolite–reaction–enzyme–gene networks. As shown in Figs. 9 and 28 metabolic enzymes associated with 5 differentially abundant metabolites were identified.

**Fig. 10:**
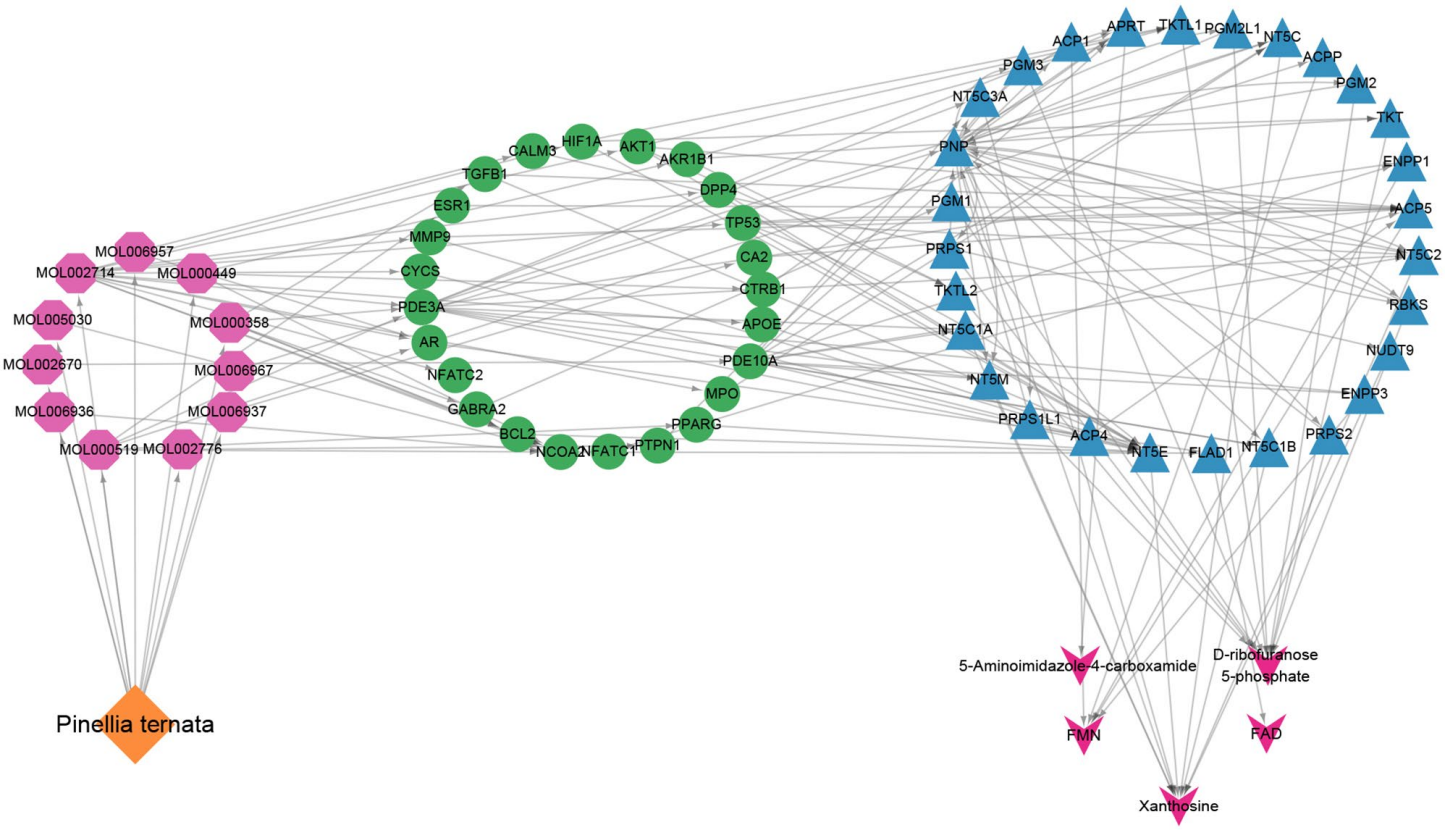
The networks of drug–bioactive component–target gene–metabolic enzyme–metabolite interactions. The orange diamond represents *Pinellia ternata*; the purple hexagonal nodes represent the candidate active components in *Pinellia ternata;* the green circular nodes represent the target genes of the active components; the blue triangle represents the key metabolic enzymes, and the purple arrow represents the key metabolites

To further investigate how the target genes of the effective components in *Pinellia ternata* regulate metabolic enzymes to differentially express metabolites, the above 99 target proteins and 28 metabolic enzymes were analyzed for protein interactions via the DAVID database. Through interrelated mapping, 24 target genes of 11 compounds in *Pinellia ternata* were found to be closely related to 28 metabolic enzymes in lung cancer cells (Fig. 10). The affected pathways were purine metabolism, riboflavin metabolism and the pentose phosphate pathway. These compounds may play essential roles in the inhibitory effect of *Pinellia ternata* on lung cancer cells.

### Western blot verification of target proteins of active components in *Pinellia ternata*

**Fig. 11:**
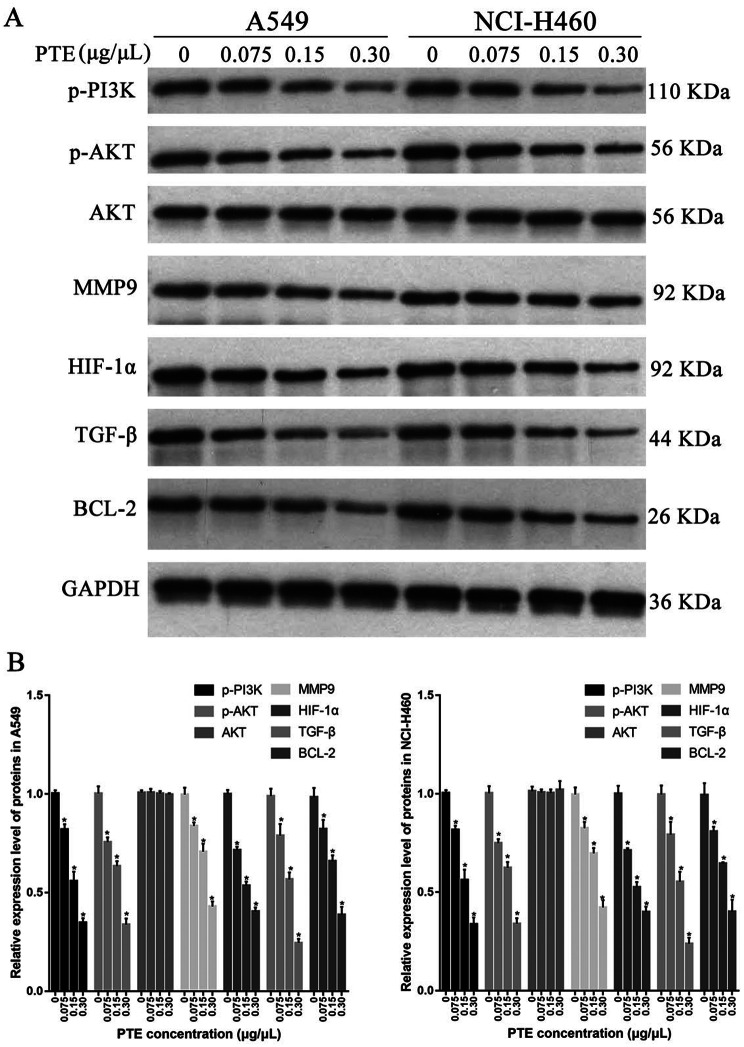
Western blot verification of target proteins of active components in Pinellia ternata. (**A**) Western blot of proteins p-PI3K p-AKT, MMP9, HIF-1α, TGF-β, BCL-2, and AKT in A549 and NCI-H460 cells, (**B**) Relative expression level of proteins p-PI3K p-AKT, MMP9, HIF-1α, TGF-β, BCL-2, and AKT in A549 and NCI-H460 cells. * represents *p* < 0.05 compared with the control group

In order to verify whether the target proteins involved in the above network are involved in inhibiting the proliferation of lung cancer cells, we carried out WB experiment to verify the expression of p-PI3K p-AKT, MMP9, HIF-1α, TGF-β, BCL-2, and AKT. As shown in Fig. 11, the extract of *Pinellia ternata* could significantly inhibit the expression of PI3K/AKT and other proteins in a concentration dependent manner, indicating that the active components of *Pinellia ternata* could target and regulate the expression of these proteins.

## Discussion

*Pinellia ternata* is a traditional Chinese medicine recorded by ancient physicians for relieving clinical diseases such as cough and sputum [27]. Our experiments showed that *Pinellia ternata* extracts could significantly inhibit the proliferation, migration, and invasion of lung cancer cells *in vitro.* By using network pharmacology methods, 13 ingredients, mainly sterols, alkaloids, flavonoids, and cyprinosides, were selected from the TCMSP database. The results of modern pharmacological research have shown that these categories of compounds play important roles in regulating tumorigenesis, suggesting that network pharmacology for screening effective active ingredients of drugs has important reference value [28]. The network of drug–key chemical component–disease targets not only revealed the progress of ancient Chinese medicine decoctions but also suggested that pinasterol, β-sitosterol, carvendine, baicalein, coniferin and other chemical components may play key regulatory roles in the targeted treatment of lung cancer. β-Sitosterol has been found in many Chinese herbal medicines to significantly inhibit the proliferation of tumor cells; specifically, in lung cancer, β-sitosterol can target and regulate Trx/Trx1 reductase to induce apoptosis in lung cancer cells [29–31]. Stigmasterol is an important component of phytosterols and is mainly isolated from soybeans and lentils. Studies have shown that it has broad-spectrum anticancer, antibacterial and antioxidant effects. It could be used to treat breast and colorectal cancer patients. Studies have shown that stigmasterol can significantly reduce the burden of metastatic tumors at cancer sites, mainly by reducing pAKT, metastasis marker genes (alkaline phosphatase, matrix metalloproteinases, epithelial to mesenchymal transcription factors), vascular growth factor (vascular endothelial growth factor), CD31 and continuous expression of cell proliferation antigen (Ki67, proliferative cell nuclear antigen) [32]. Baicalein has broad-spectrum physiological activities, such as antibacterial, antiviral, and inflammatory effects, and can exert antitumor effects through multiple targets and multiple pathways [33, 34]. Carvendine has obvious antitumor effects due to its anti-inflammatory, immune-regulating and antiviral effects. It mainly acts on potential targets, such as the efferent nervous system, ion channels, PDE10A, and coagulation factors [35]. However, these studies still lack research on the antitumor functions of other components and have not systematically identified which components participate in the regulatory mechanisms affecting the malignant phenotype characteristics of lung cancer cells from the perspective of metabolic pathways.

Researchers are increasingly relying on metabolomics to explore disease mechanisms and intervention strategies. We identified 5 significant metabolites of *Pinellia ternata* that act against lung cancer cells, as well as their related metabolic pathways. However, given the complexity and heterogeneity of metabolomics, data analysis and interpretation are collaborative efforts [36]. Network pharmacology is a systems biology-based methodology [37]. It evaluates drug polypharmacological effects at the molecular level to predict the interaction of natural products and proteins as well as to determine the major underlying mechanisms [38]. Network pharmacology can further validate the therapeutic regulation of metabolic networks and facilitate the identification of key targets and biomarkers [39]. In this study, network pharmacology greatly improved the screening of metabolites of *Pinellia ternata* against lung cancer and elucidated the underlying mechanisms of action. By combining metabolomics with network pharmacology, 11 bioactive compounds, 24 key targets, 28 metabolic enzymes and 5 metabolites (D-ribose 5-phosphate, FAD, FMN, 5-amino-4-imidazolecarboxyamide and xanthosine) and 3 related pathways (purine metabolism, riboflavin metabolism and the pentose phosphate pathway) were identified. This strategy provides a suitable method to verify the results of the two approaches. It is also practical to screen for metabolites and targets of other natural compounds.

Purine metabolism represents a potential therapeutic pathway in cancer therapy. Purine, an abundant substrate in organisms, is a critical raw material for cell proliferation and an important factor for immune regulation [40]. The purine *de novo* pathway and salvage pathway are tightly regulated by multiple enzymes, and dysfunction of these enzymes leads to excessive cell proliferation and immune imbalance that results in tumor progression [41, 42]. For example, inosine strongly enhances the proliferation of human melanoma cells [43], and an altered ratio of adenosine to inosine has been widely observed in cancer cells, affecting growth, invasiveness, and metastasis [44]. Moreover, purines serve as potent modulators of immune cell responses and cytokine release via various receptor subtypes, such as P2X ligand-gated ion channels and G protein-coupled P2Y receptors [45], which are substantially involved in oncogenesis and tumorigenesis [46, 47]. Xanthosine is catalyzed by the substrate xanthine or xanthosine 5’-phosphate through the activity of purine-nucleoside phosphorylase or 5’-nucleotidase. 5-Aminoimidazole-4-carboxamide is synthesized by the substrate 1-(5’-phosphoribosyl)-5-aminoimidazole-4-carboxamide through the activity of adenine phosphoribosyltransferase [48]. The levels of xanthosine and 5-aminoimidazole-4-carboxamide increased in PTE-treated lung cancer cell groups. Studies have shown that the administration of xanthosine did not affect the proportion of epithelial stem cells in bovine breast tissue but had potential negative effects on cell proliferation, and tumor development in mice was also limited by xanthosine administration [49]. Studies have also indicated that 5-aminoimidazole-4-carboxamide riboside combined with methotrexate has synergistic anticancer effects on human breast cancer and hepatocellular carcinoma [50]. At present, there is almost no literature on the involvement of xanthosine and 5-aminoimidazole-4-carboxamide metabolites in cell death and migration, but there are relevant reports on the involvement of purine metabolic pathways in cell migration mechanisms [51]. Therefore, *Pinellia ternata* may inhibit the proliferation of lung cancer cells through purine metabolism, especially by altering the metabolic levels of xanthosine and 5-aminoimidazole-4-carboxamide.

Riboflavin metabolism is closely related to human health. Riboflavin is an essential micronutrient for normal cellular activity, and riboflavin deficiency may result in disease, such as cancer. FMN synthesizes FAD through the activity of FAD synthetase, or FAD synthesizes FMN through the activity of nucleotide diphosphatase [52]. A high level of spontaneous intracellular FAD fluorescence is an indicator of cell pathology and indicates subsequent apoptosis and necrosis [53]. In this study, the levels of FAD and FMN increased in PTE-treated lung cancer cell groups. Many retrospective clinical studies have shown a close correlation between riboflavin deficiency and tumor development [54, 55]. In vitro experiments have demonstrated that riboflavin depletion promotes tumorigenesis in HEK293T and NIH3T3 cells by sustaining cell proliferation and regulating cell cycle-related gene transcription [56]. Riboflavin supplementation has been shown to be an adjuvant for the treatment of tumors [57]. Moreover, dietary riboflavin supplementation can reduce the risk of cancer in clinical practice [58]. All these studies suggested that *Pinellia ternata* may inhibit the proliferation of lung cancer cells by promoting riboflavin metabolism.

The pentose phosphate pathway (PPP) is a major pathway for glucose catabolism [59]. It has become clear that the PPP plays a critical role in regulating cancer cell growth by supplying cells with not only ribose-5-phosphate but also NADPH for the detoxification of intracellular reactive oxygen species, reductive biosynthesis and ribose biogenesis. Thus, alteration of the PPP contributes directly to cell proliferation, survival and senescence [60]. Many studies have shown that inhibiting the PPP can inhibit the development of tumors [61, 62], but there are currently almost no reports on the regulation of cell death and migration by D-ribouranose 5-phosphate. Interestingly, in this study, the lung cancer cell group treated with *Pinellia ternata* showed a decrease in D-ribofuranose 5-phosphate expression. D-Ribofuranose 5-phosphate is an important intermediate metabolite of the pentose phosphate pathway and can be synthesized mainly through the metabolism of the substrate D-ribose through the activity of ribokinase. Therefore, *Pinellia ternata* may inhibit the proliferation of lung cancer cells by inhibiting the PPP pathway.

## Conclusion

In this study, we first demonstrated that *Pinellia ternata* inhibited the proliferation, migration, and invasion of lung cancer cells. Subsequently, 5 key metabolites and 3 important metabolic pathways were identified through cell metabolomics screening. Combined with network pharmacology, we identified 11 effective active components, and an association network of *Pinellia ternata*–bioactive component–target gene–metabolic enzyme–metabolite interactions was constructed. This is the first development of a new comprehensive strategy based on metabolomics and network pharmacology to explore key targets and mechanisms of *Pinellia ternata* in the treatment of lung cancer. This study provides data and theoretical support for in-depth research on its mechanism of action, laying the foundation for clinical application. Further systematic molecular biology experiments are needed to verify the exact mechanism involved. This study also provides a new paradigm for determining the potential mechanisms of the pharmacological effects of natural compounds.

### Electronic supplementary material

Below is the link to the electronic supplementary material.

Supplementary Material 1

Supplementary Material 2

Supplementary Material 3

Supplementary Material 4

Supplementary Material 5

Supplementary Material 6

Supplementary Material 7

Supplementary Material 8

Supplementary Material 9

Supplementary Material 10

Supplementary Material 11

Supplementary Material 12

Supplementary Material 13

## Data Availability

The data that support the findings of this study are owned by the authors and/or no permissions are required.
