# Supplementary material for: Integrated network pharmacology and metabolomics to reveal the mechanism of Pinellia ternata inhibiting non-small cell lung cancer cells
Source: BMC Complement Med Ther. 2024 Jul 11;24:263. doi: 10.1186/s12906-024-04574-3 (PMC11238457; doi:10.1186/s12906-024-04574-3)
Supplement: Supplementary file 1 — Supplementary Material 1 [file 12906_2024_4574_MOESM1_ESM.doc]

**Table S1 Basic information of 13 bio-active compounds in *Pinellia ternata***

Number Molecular Name OB (%) DL Molecules structure Herb


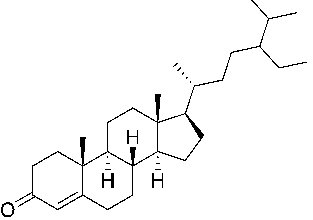


MOL1755 24-Ethylcholest- 36.08 0.76 Pinellia

4-en-3-one


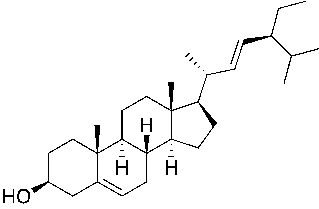


MOL449 Stigmasterol 43.83 0.76 Pinellia


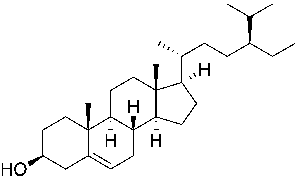


MOL358 beta-sitosterol 36.91 0.75 Pinellia


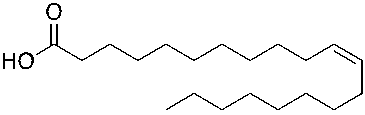


MOL5030 gondoic acid 30.70 0.20 Pinellia


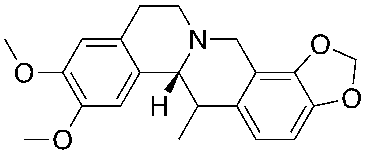


MOL2670 Cavidine 35.64 0.81 Pinellia


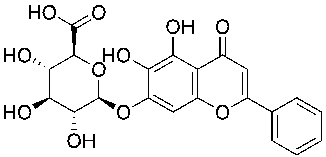


MOL2776 Baicalin 40.12 0.75 Pinellia


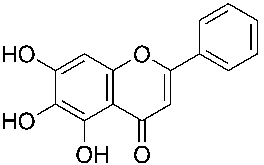


MOL2714 baicalein 33.52 0.21 Pinellia


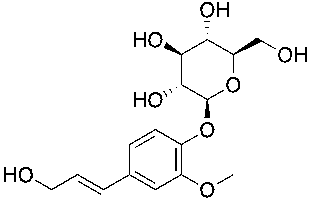


MOL519 coniferin 31.11 0.32 Pinellia


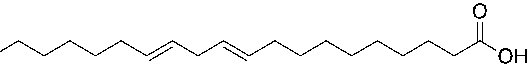
MOL6936 10,13-eicosadienoic 39.99 0.20 Pinellia


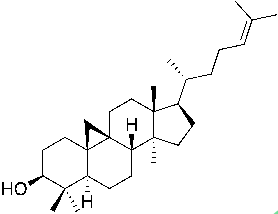


MOL3578 Cycloartenol 38.69 0.78 Pinellia


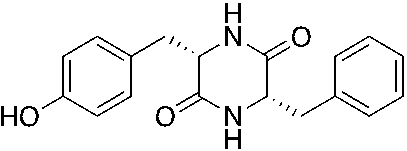


MOL6957 (3S,6S)-3-(benzyl)- 46.89 0.27 Pinellia

6-(4-hydroxybenzyl)piperazine-

2,5-quinone


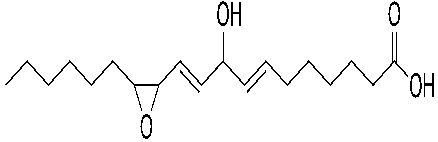


MOL6937 12,13-epoxy-9- 42.15 0.24 Pinellia

hydroxynonadeca-


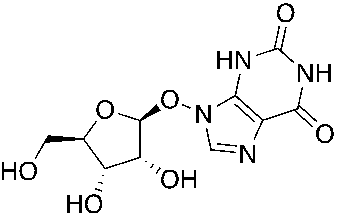
7,10-dienoic acid

MOL6967 beta-D-Ribofuranoside 44.72 0.21 Pinellia
