# Supplementary figures and images for: Integrated network pharmacology and metabolomics to reveal the mechanism of Pinellia ternata inhibiting non-small cell lung cancer cells
Source: BMC Complement Med Ther. 2024 Jul 11;24:263. doi: 10.1186/s12906-024-04574-3 (PMC11238457; doi:10.1186/s12906-024-04574-3)

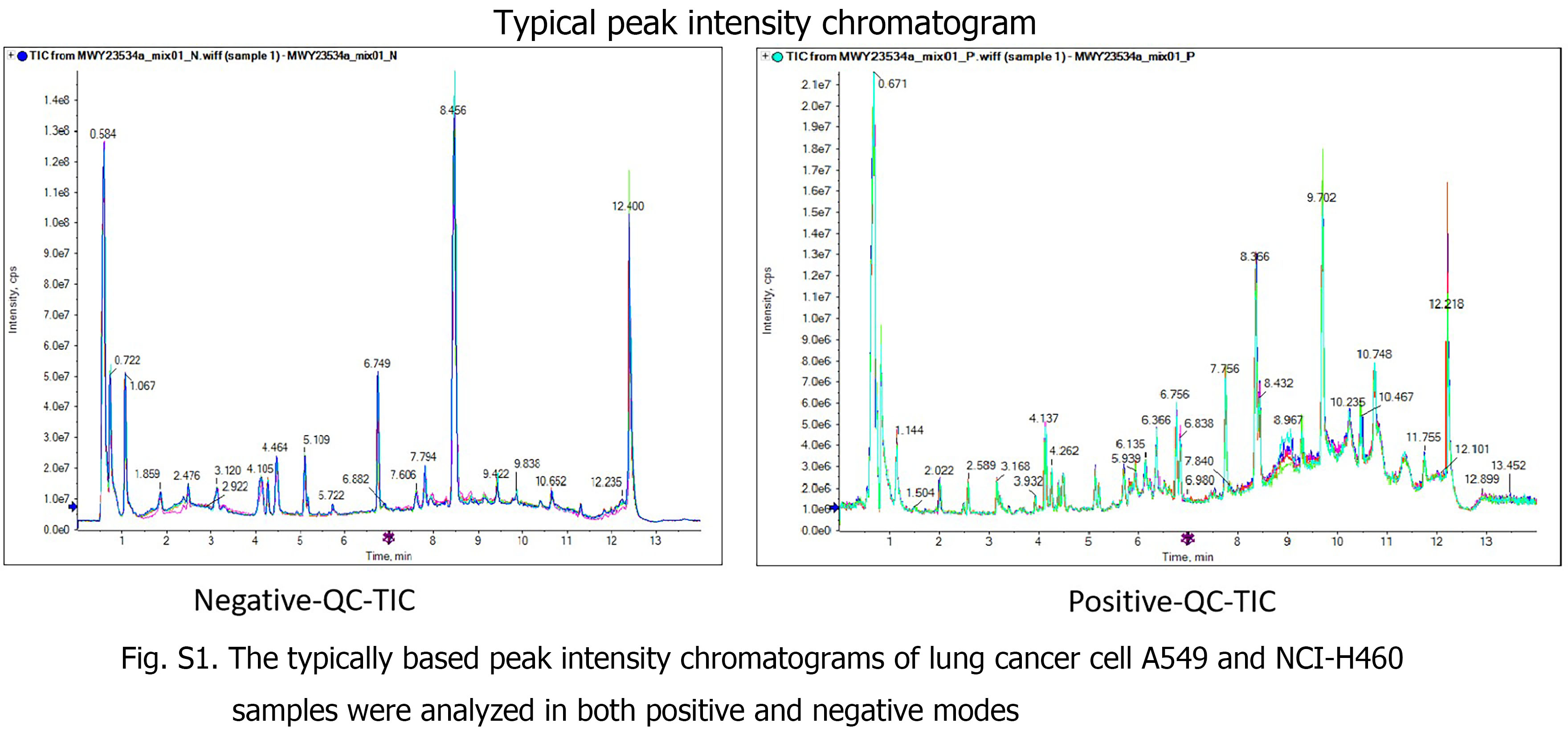

Supplement: Supplementary file 2 — Supplementary Material 2 [file 12906_2024_4574_MOESM2_ESM.jpg]

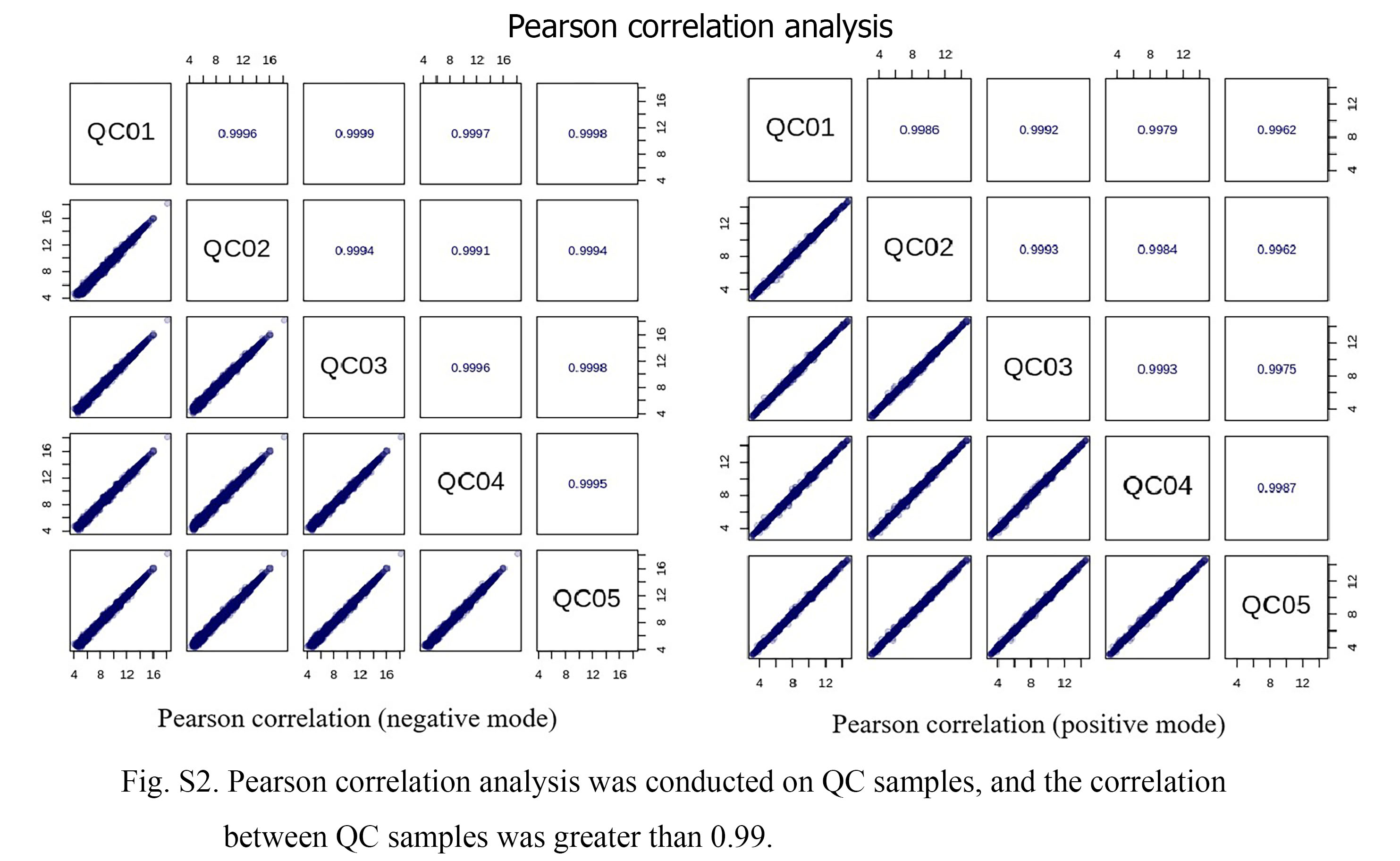

Supplement: Supplementary file 3 — Supplementary Material 3 [file 12906_2024_4574_MOESM3_ESM.jpg]

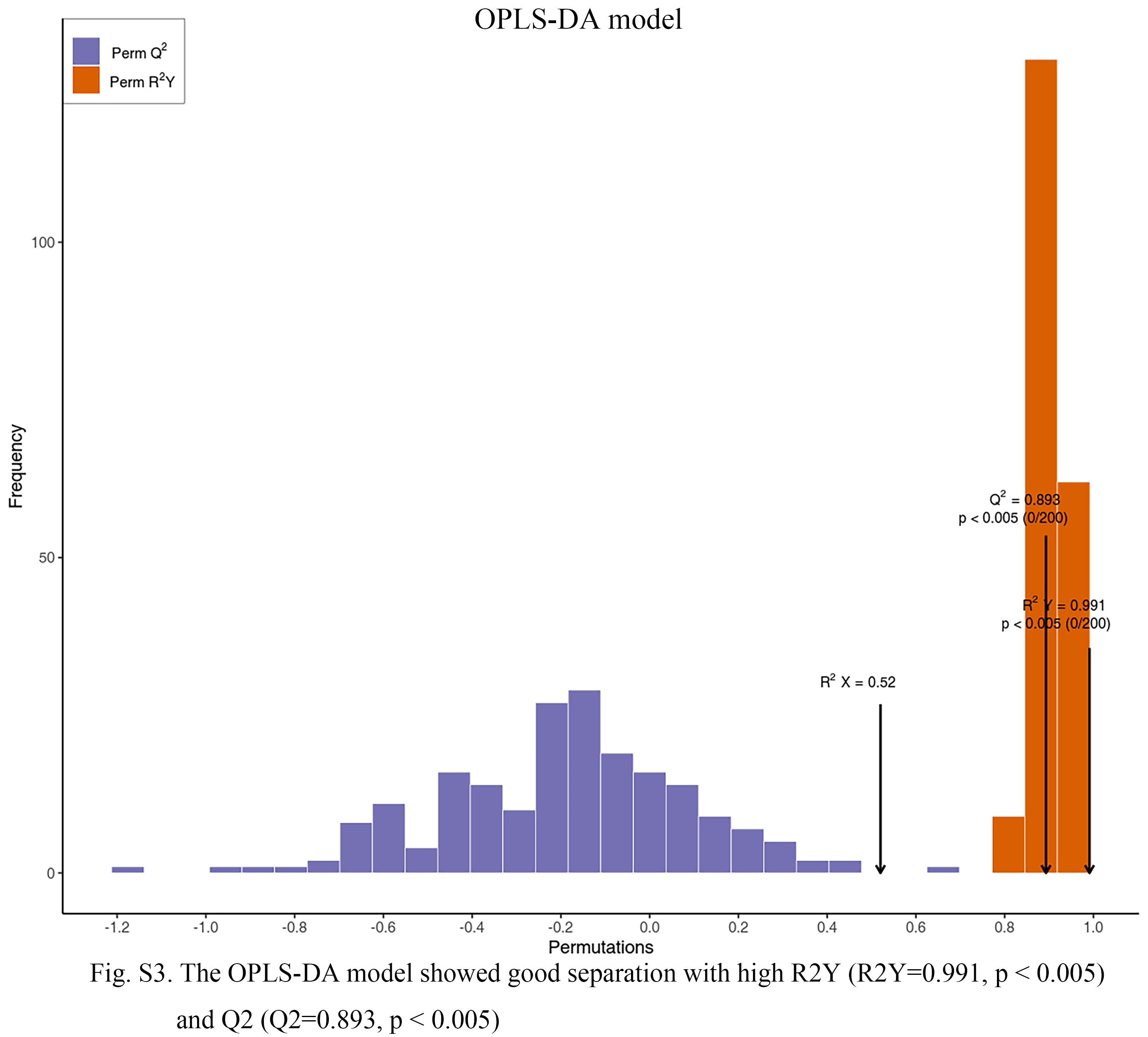

Supplement: Supplementary file 4 — Supplementary Material 4 [file 12906_2024_4574_MOESM4_ESM.jpg]

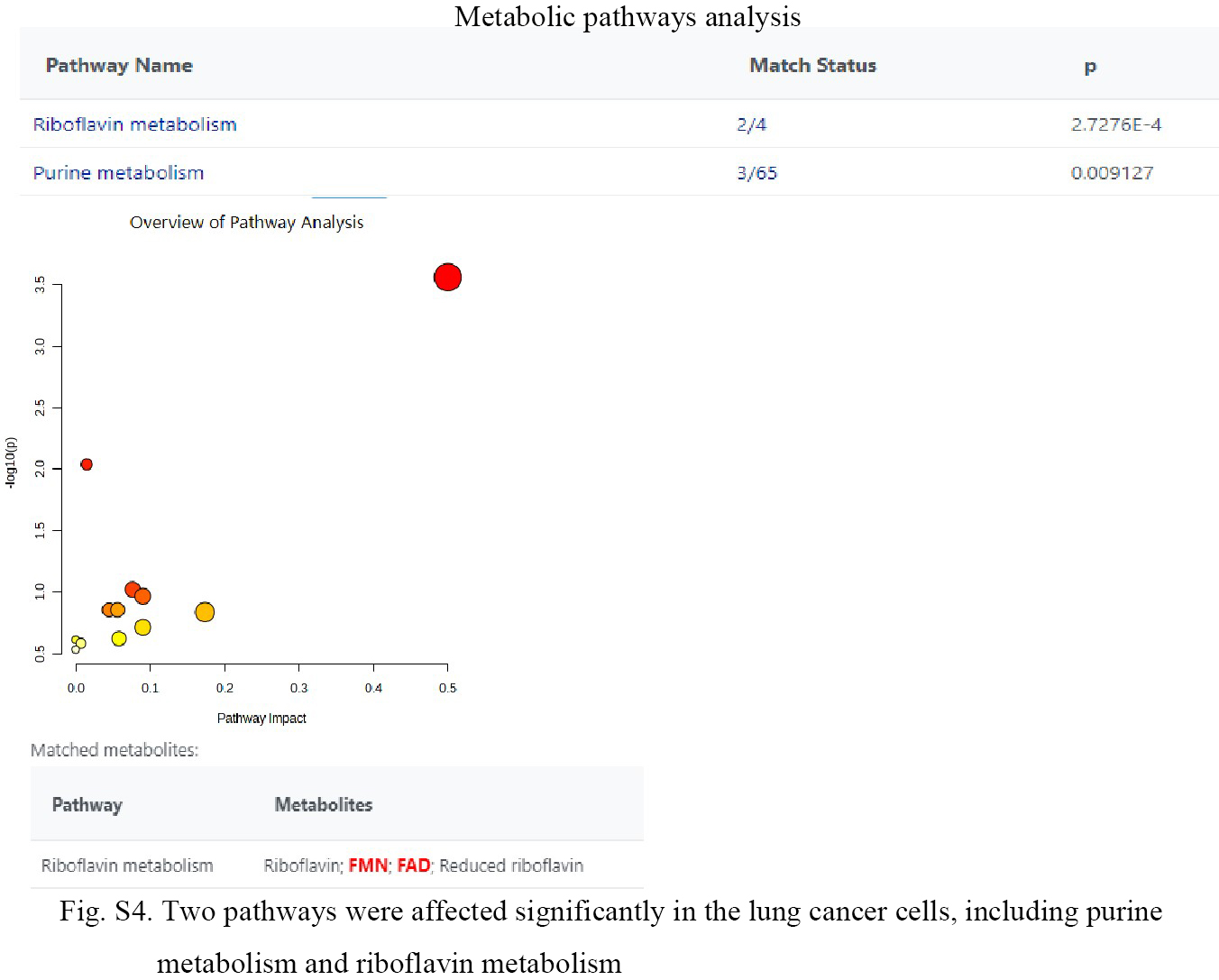

Supplement: Supplementary file 5 — Supplementary Material 5 [file 12906_2024_4574_MOESM5_ESM.jpg]

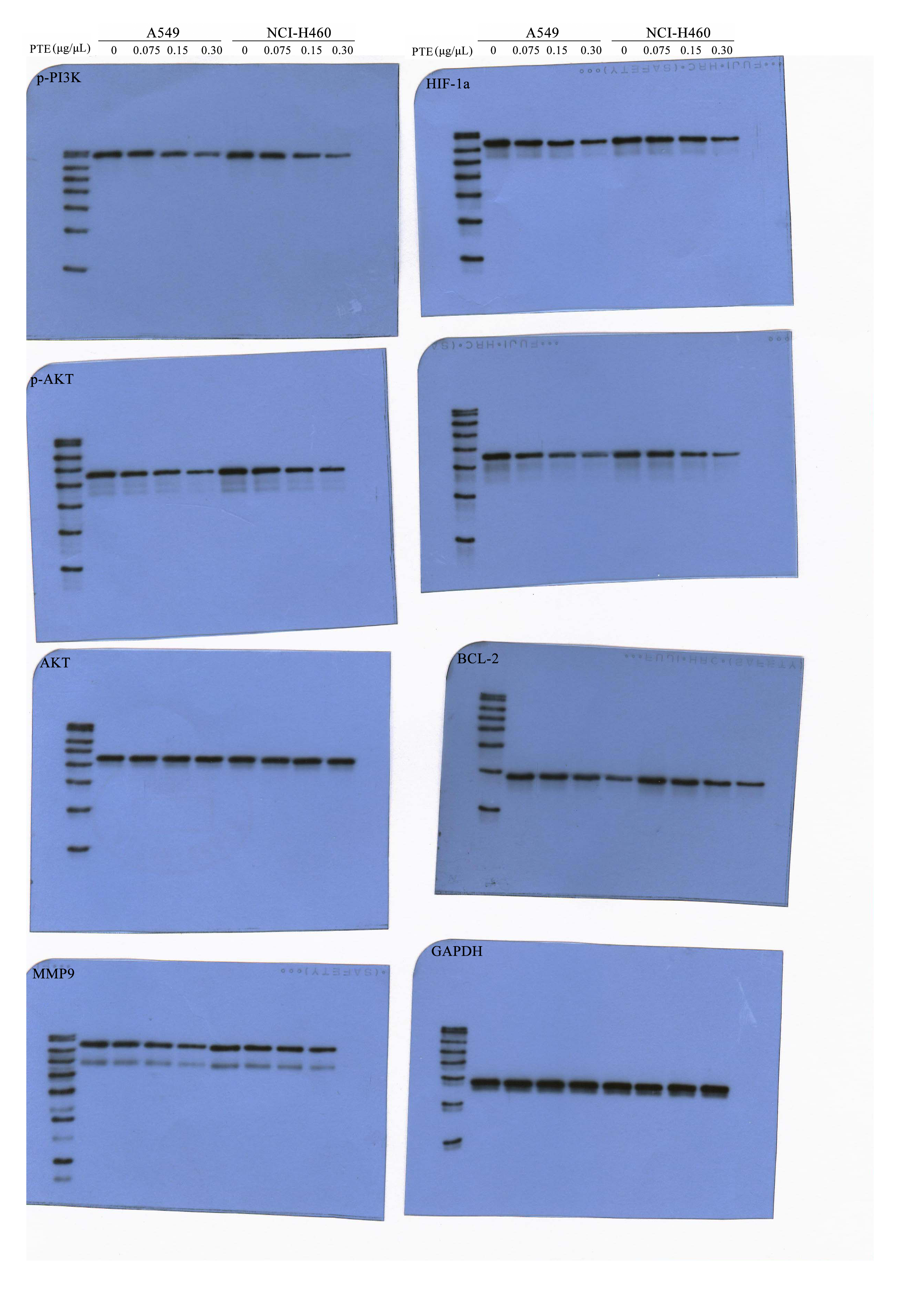

Supplement: Supplementary file 13 — Supplementary Material 13 [file 12906_2024_4574_MOESM13_ESM.tif]
